# Common Cerambycid Pheromone Components as Attractants for Longhorn Beetles (Cerambycidae) Breeding in Ephemeral Oak Substrates in Northern Europe

**DOI:** 10.1007/s10886-019-01082-4

**Published:** 2019-06-28

**Authors:** Mikael A. Molander, Inis B. Winde, Joseph Burman, Franklin N. Nyabuga, Tobias U. T. Lindblom, Lawrence M. Hanks, Jocelyn G. Millar, Mattias C. Larsson

**Affiliations:** 1 Unit of Chemical Ecology, Department of Plant Protection Biology, Swedish University of Agricultural Sciences, Box 102, Sundsvägen 14, 230 53 Alnarp, Sweden; 2 Present Address: Ecology Research Group, Canterbury Christ Church University, North Holmes Road, Canterbury, Kent, CT1 1QU United Kingdom; 3 Present Address: Department of Biological Sciences, University of Embu, P.O. Box 6, 60100 Embu, Kenya; 4 Present Address: Department of Crop Production Ecology, Swedish University of Agricultural Sciences, Box 7043, 750 07 Uppsala, Sweden; 5 Department of Entomology, University of Illinois at Urbana-Champaign, Urbana, IL 61801 USA; 6 Departments of Entomology and Chemistry, University of California, Riverside, CA 92521 USA

**Keywords:** Semiochemical, (*R*)-2-Methyl-1-butanol, (*R*)-3-Hydroxy-2-hexanone, Monitoring, Biodiversity, Red List

## Abstract

Longhorn beetles are ecologically important insects in forest ecosystems as decomposers of woody substrates, microhabitat engineers, and as components of forest food webs. These species can be greatly affected both positively and negatively by modern forestry management practices, and should be monitored accordingly. Through headspace sampling, coupled gas chromatography-electroantennography, gas chromatography-mass spectrometry, and field bioassays, we identified two compounds, 2-methyl-1-butanol and 3-hydroxy-2-hexanone, that constitute aggregation-sex pheromone attractants of three cerambycid species which breed primarily in different types of fresh, recently dead oak wood in Northern Europe: *Pyrrhidium sanguineum* (L.), *Phymatodes alni* ssp*. alni* (L.), and *Phymatodes testaceus* (L.) (Cerambycinae: Callidiini). Analyses of headspace volatiles collected from live insects indicated that the male-produced aggregation-sex pheromone of *P. sanguineum* is a 1–15:100 blend of (*R*)-2-methyl-1-butanol and (*R*)-3-hydroxy-2-hexanone, whereas the corresponding ratios for *P. alni* were 70–110:100. In field bioassays, adult *P. sanguineum* and *P. alni* were significantly attracted to multiple blends with varying ratios of the two compounds. When tested individually, the compounds were minimally attractive. In contrast, adult *P. testaceus* exhibited nonspecific attraction to both of the individual compounds and to different blends, despite the hydroxyketone not being part of its pheromone, which consists of (*R*)-2-methyl-1-butanol alone. Overall, our results suggest that a blend of 50:100 of racemic 2-methyl-1-butanol and 3-hydroxy-2-hexanone is appropriate for parallel, cost-efficient pheromone-based monitoring of all three species. In particular, these species could serve as useful indicators of how modern forestry practices affect a whole guild of saproxylic insects that require ephemeral deadwood substrates for successful breeding.

**Electronic supplementary material:**

The online version of this article (10.1007/s10886-019-01082-4) contains supplementary material, which is available to authorized users.

## Introduction

Longhorn beetles (Cerambycidae) constitute important components of forest ecosystems worldwide, where they contribute to the decomposition of dead wood, create microhabitats for other organisms, and are important components of forest food webs (Buse et al. 2008; Hogstad and Stenberg 1997). Longhorn beetles typically breed in dying or recently dead wood, but their larval habitats may also include healthy living wood, as well as decaying above- or below-ground wood, and the stems of living herbs and bushes (Linsley 1959). Some longhorn beetles are economically important pests in forestry or agriculture, with high potential to become invasive pest species (Haack 2017). As with many other insect taxa, the potential of some species to become pests has provided the primary motivation to identify pheromones for both endemic and invasive longhorn beetles (Allison et al. 2004; Hanks and Millar 2016).

In the last decade, the pheromones of a small number of insect species have also been exploited for conservation purposes, with pheromone-baited traps being used to monitor the presence of rare and threatened species that are otherwise difficult to survey (Larsson 2016). This includes some longhorn beetles, such as species from the genera *Prionus* (Barbour et al. 2011), *Tragosoma* (Ray et al. 2012), *Desmocerus* (Ray et al. 2014), and *Rosalia* (Žunič Kosi et al. 2017). Overall, there is an enormous untapped potential to use longhorn beetles as indicator species in forestry and forest biodiversity conservation, by means of pheromone-based monitoring. Many species are currently in decline due to the scarcity of deadwood resources in modern managed forests and in agricultural landscapes (Jeppsson et al. 2010; Lindhe et al. 2010). Still other species that are not themselves directly threatened at present constitute important food resources for other threatened organisms such as woodpeckers (Hogstad and Stenberg 1997), and could therefore be useful for monitoring the general state of forest food webs at local and landscape levels. This includes a whole guild of longhorn beetles that breed in ephemeral woody substrates such as fresh, recently killed trunks, stumps, and branches, which are only suitable as larval substrates for short periods of time, typically 1–2 years (Ehnström and Holmer 2007; Linsley 1959).

Longhorn beetles breeding in ephemeral fresh woody substrates have high potential to reflect relatively fast dynamic processes typical of managed forest ecosystems. They thus represent a complementary conservation target to the saproxylic insects that breed in old trees or very coarse, late-successional woody materials, and which are a major focus of current deadwood conservation efforts (e.g., Milberg et al. 2016; Ranius and Jansson 2000; Seibold et al. 2015). Their ability to exploit relatively young and quickly regenerating deadwood resources would seemingly make these longhorn beetles unlikely targets of conservation concern. However, in modern forestry, even early successional species may suffer from declining breeding substrates due to the efficient harvesting of residue materials from logging as biofuel resources, which could make even small woody debris such as twigs and branches a limiting resource in modern production forests. Of equal or even greater concern might be the removal of woody debris for biofuel after colonization by woodborers, so that the host resource becomes a population sink for these species (Hedin et al. 2008).

The cerambycid *Pyrrhidium sanguineum* (L.) (Cerambycinae: Callidiini) is a species representative of the guild of longhorn beetles described above. It has a relatively narrow substrate range and primarily breeds in fresh, recently dead, medium-sized logs and branches of oak (Ehnström and Holmer 2007). Although not threatened from a global or European perspective, *P. sanguineum* is considered a rare species in Northern Europe, including Sweden, where it is nationally red listed as Near Threatened (ArtDatabanken 2015), and Denmark, where it is red listed as Vulnerable (Wind and Pihl 2010). It could thus have high potential to function as an indicator species of scarce ephemeral deadwood resources, provided it can be monitored efficiently with pheromone traps. The pheromone of *P. sanguineum* had previously been reported as a blend of (*R*)-3-hydroxy-2-hexanone (main component), (2*S*,3*R*)-2,3-hexanediol and (2*R*,3*R*)-hexanediol, that was significantly attractive to unmated females in wind tunnel assays (Fettköther 1995; Schröder et al. 1994). However, to our knowledge no field trapping studies have demonstrated attraction of *P. sanguineum* to a blend of these compounds.

Here we summarize research that reinvestigated the composition of the aggregation-sex pheromone of *P. sanguineum* by collection of headspace odors from live beetles, and analysis of the resulting extracts by gas chromatography-electroantennographic detection (GC-EAD) and coupled gas chromatography-mass spectrometry (GC-MS), followed by field bioassays of possible pheromone components. Similar to previous work, our experiments confirmed that (*R*)-3-hydroxy-2-hexanone was the main pheromone component of the species, but we also found that (*R*)-2-methyl-1-butanol is a previously unreported minor pheromone component, and that the blend of these two compounds constitutes the male-produced aggregation-sex pheromone of *P. sanguineum*. We also demonstrate that the same compounds function as the aggregation-sex pheromone of the closely related species *Phymatodes alni* ssp*. alni* (L.) (henceforth referred to as *P. alni*). Further, we observed that a third species, *Phymatodes testaceus*, was significantly attracted to both 3-hydroxy-2-hexanone and 2-methyl-1-butanol as single compounds, and to blends of these, although the species’ pheromone has previously been identified as (*R*)-2-methyl-1-butanol alone (Hanks et al. 2019).

## Materials and Methods

### Collection of Insects for Experiments

In the spring of 2011 and 2012 freshly cut branches of pedunculate oak (*Quercus robur* L.)*,* were placed at two sites in southeastern Sweden, one site within Ecopark Hornsö in Kalmar County (decimal degrees: 57.0280 N, 16.1638 E) and one site at Gö nature reserve in Blekinge County (DD: 56.1273 N, 15.3092 E). The fresh substrates were intended to attract females of early successional longhorn beetles that are dependent on this substrate for oviposition. The wood material was collected in the autumn of each year and brought back to the Alnarp Campus to rear out any longhorn beetles. Additional oak substrates (twigs and branches) were taken from a stack of biofuel material in the winter of 2015 at Hornsö Ecopark (DD: 57.0202 N, 16.1702 E).

The dead wood was stored outdoors until it was brought inside in portions during late winter and held in ventilated plastic containers or fine mesh cages in the greenhouse facilities to collect emerging adult beetles. Adults of *P. sanguineum* primarily emerged from the wood collected in 2011 and 2012, i.e., from medium-sized branches ~5–15 cm in diameter, whereas *P. alni* only emerged from thin twigs (~1–3 cm in diameter) collected in 2015. In total, >600 *P. sanguineum* and 341 *P. alni* emerged. Randomly selected subsets of ~35 individuals of each species were used for collection of headspace volatiles (see below).

The beetles were sexed and males and females were held separately in plastic jars containing pieces of fresh oak branches and water-soaked pieces of paper to provide moisture, either in a refrigerator at ~8 °C or in a climate controlled chamber at 25 °C, 70% RH, with a 12.5:11.5 L/D cycle (8:30 AM to 9 PM). Males and females of *P. sanguineum* were easily distinguished by the relative length of the antennae. The antennae of males extend beyond the tip of the abdomen, while the antennae of females do not reach the tip of the abdomen (Hansen 1966). To sex the much smaller species *P. alni*, we placed pairs of beetles in small containers and observed if copulation was attempted. Normally, when a male and female were placed together, copulation was initiated immediately after the male first touched the female with his antennae. Pairs that attempted copulation were quickly separated and transferred to a plastic jar containing other individuals of the same sex. Individuals that made physical contact but did not attempt to copulate were excluded, as were beetles that had not made contact after ~0.5 min.

### Collection of Insect-Produced Volatiles

Volatile compounds from *P. sanguineum* and *P. alni* were collected from the headspace of beetles held in 200 ml Pyrex^®^ glass bottles with plastic lids sealed with Teflon^®^ lining. Males and females were held in separate bottles (and an empty control bottle was also used for *P. alni*, but not for *P. sanguineum*). Two holes were drilled through each lid to accommodate air inlets and outlets. Before collection, the bottles and lids were rinsed sequentially with water, ethanol, and acetone and allowed to dry overnight. Air was drawn through each bottle at a rate of ~0.2 l/min using an air pump (model PM 10879 NMP 03, KNF Neuberger, Freiburg, Germany). Incoming air was purified by passage through a bed of granulated charcoal in a Teflon^®^ tube, held in place with polypropylene wool plugs (Supelco/Sigma-Aldrich, Munich, Germany) and short pieces of smaller Teflon^®^ tubing (length 2 mm, inner ø 1.5 mm), inserted into the main column on both sides of the adsorbent material. Air was pulled from the bottle through a bed of 25 mg Porapak™ Q (mesh size 50–80, Supelco/Sigma-Aldrich) in a Teflon^®^ tube (length 50 mm, inner ø 3 mm) and secured as already described. Both the charcoal filters for incoming air and the collectors were rinsed thoroughly with hexane and acetone, and then air-dried to allow the solvent to evaporate before use. Groups of 4 to 10 male and female beetles were put in separate bottles, and collections were run in a climate-controlled chamber at 25 °C for 4–6 h between 10 AM and 6 PM, which encompassed the normal diel activity period of the two species (MAM, pers. obs.). After aeration, the beetles were released back into the containers with each sex. Thus, mixtures of new and previously aerated individuals were used in subsequent collections. A total of five series of collections with *P. sanguineum* and two collections with *P. alni* were performed, with each series generating a sample from males and females respectively (and for *P. alni* also a blank control sample). Trapped volatiles were eluted from collectors with 2 × 150 μl of redistilled hexane, and samples were stored in glass vials at −18 °C until used for analyses.

### Electrophysiology

Antennal responses of male and female *P. sanguineum* to the headspace samples from both sexes were recorded using coupled gas chromatography-electroantennographic detection (GC-EAD). Two μl of the aeration samples were injected into a GC (model 7890A, Agilent Technologies, Palo Alto, CA, USA) in splitless mode with an injector temperature of 225 °C (split vent opened after 0.5 min). The GC was fitted with a DB-WAX capillary column (30 m × 0.25 mm inner ø, d.f. 0.25 μm; J&W Scientific, Folsom, CA, USA), and eluting compounds were detected with a flame ionization detector (FID). Carrier gas was hydrogen at a constant flow rate of 2.1 ml/min. The GC oven was programmed from 30 °C, with a 3 min hold, and then increased at 20 °C/min to 225 °C, and was held for 10 min. The column effluent was split with a 3D/2 low dead volume four-way-cross (Gerstel, Mülheim, Germany), 27.6 kPa of nitrogen was added through the extra arm, and the resulting effluent was split 1:1 between the FID and the EAD. The GC effluent capillary for the EAD passed through a transfer line (ODP-3, Gerstel), which tracked the GC oven temperature, into a glass tube (30 cm length × 0.8 cm inner ø), where it was diluted with a charcoal-filtered, humidified airstream (1.0 L/min).

Antennae were excised using microscissors at the first antennal segment near the head of the beetle and mounted with electrode gel (article 1330, CefarCompex, Malmö, Sweden) onto a forked electroantennographic multi-probe (Syntech, Kirchzarten, Germany). The complete beetle antenna was positioned 0.5 cm from the outlet of the effluent tube. Signals from the antenna and the GC-FID were recorded simultaneously with a Syntech IDAC-2 digital converter and Syntech GCEAD 3.1 software. Male-produced extracts were run with antennae from 7 males and 10 females, and female-produced extracts were run with antennae from 7 males and 6 females. All extracts of volatiles from males and females of *P. sanguineum* were analyzed with GC-EAD, using at least one antenna for each extract.

### Identification of Potential Pheromone Components

After detection of electrophysiologically active compounds at Alnarp Campus, headspace extracts from *P. sanguineum* were analyzed by GC-MS at UC Riverside on a medium polarity DB-17 column (30 m × 0.25 mm inner ø, d.f. 0.25 μm; J&W Scientific) mounted in a Hewlett-Packard 6890 GC (HP, now Agilent Technologies) coupled to an HP 5973 MS. The MS was operated in electron impact ionization mode (EI, 70 eV), with a scan range of 40–400 *m/z*, and transfer line temperature of 250 °C. Sample aliquots (1 μl) were injected in splitless mode (injector temp 250 °C, split vent opened after 0.5 min), with the oven programmed from 40 °C for 1 min, then 10 °C/min to 280 °C, hold for 10 min. Helium carrier gas was used (linear flow rate, 37 cm/s). Injections were initially made with an injector temp of 250 °C, and then repeated with a temp of 125 °C to minimize isomerization of the thermally labile hydroxyketones.

The absolute configurations of beetle-produced compounds were determined by analyses of extracts on a chiral stationary phase Cyclodex B GC column (30 m × 0.25 mm inner ø, d.f. 0.25 μm; J&W Scientific). Samples were injected split (split ratio ~20:1), with a head pressure of 172 kPa. The oven was programmed from 50 °C for 1 min or in some analyses for 2-methyl-1-butanol for 5 min, then 3 °C per min to 220 °C, hold for 10 min. The injector temperature was set at 150 °C to minimize isomerization of the hydroxyketones. Samples were injected alone, and then admixed with the racemates of authentic standards to determine which of the two peaks from the racemate was increased by the insect-produced enantiomer.

In 2015, when volatiles were collected from *P. alni*, extracts of this species were first analyzed by GC-MS at Alnarp and then with the same methods as described above at UC Riverside. The GC-MS analyses of extracts from males and females at Alnarp were performed by injecting 2 μl of each sample in splitless mode onto a 6890N GC interfaced to a 5975 MS (both Agilent Technologies). The GC was fitted with an HP-5 ms capillary column (60 m × 0.25 mm inner ø, d.f. 0.25 μm, Agilent Technologies). Helium was the carrier gas at a constant flow rate of 1.8 ml/min. The injector temperature was 225 °C (split vent opened after 0.5 min) and transfer line temperature was 150 °C. The inlet pressure was 172 kPa and the oven temperature was initially held at 30 °C for 3 min, thereafter increasing by 8 °C/min to 260 °C, with a 10 min hold. The mass spectrometer was set with a 7 min solvent delay and a scan range of 29–400 *m/z*. Spectra were taken in EI mode at 70 eV.

Sex-specific compounds were recognized by visual comparison of the chromatograms from males and females, and by using the overlay function in the Agilent ChemStation software. Tentative identification of the potential pheromone compounds was performed by matching their mass spectra to those in database libraries (NIST05, NIST11, and Wiley275) and/or by comparison with retention times and mass spectra of authentic standards. Identifications were then confirmed by injections of standards (except 2,3-hexanedione for *P. alni*). For *P. sanguineum*, relative ratios of pheromone compounds were calculated based on peak integration data from FID chromatograms from the GC-EAD analyses, whereas peak integration data from the total ion chromatograms from GC-MS analyses was used for *P. alni* (Alnarp laboratory). In the extracts from *P. alni*, we also observed that the peak of 3-hydroxy-2-hexanone included 2-hydroxy-3-hexanone when analyzed at the Alnarp campus, but subsequent analysis at Riverside with lower analysis temperatures showed that this was likely due to thermal rearrangement and that a representative insect extract contained only a trace of 2-hydroxy-3-hexanone.

### Sources of Chemicals

Samples of (*R*)- and (*S*)-3-hydroxy-2-hexanone for comparison with the insect-produced compounds were available from previous work (Lacey et al. 2007). Racemic 2-methyl-1-butanol and its (*S*)-enantiomer, and 2,3-hexanedione were purchased from Aldrich Chemical Co. (Milwaukee, WI, USA), and (*R*)-2-methyl-1-butanol was prepared by reduction of (*R*)-2-methylbutanoic acid as described in Hanks et al. (2018). 1-Hexanol (reagent grade 98%, CAS 111–27-3, Sigma-Aldrich, Hamburg, Germany) and racemic 2-methyl-1-pentanol (99.8%, CAS 105–30-6, Dr. Ehrenstorfer GmbH, Augsburg, Germany) were purchased to confirm the identities of trace compounds in extracts from *P. alni*. For the bioassays, we purchased racemic 2-methyl-1-butanol (≥ 99%, CAS 137–32-6, Sigma-Aldrich, Hamburg, Germany) and racemic 3-hydroxy-2-hexanone (CAS 54123–75-0, Bedoukian Research, Danbury, CT, USA).

### Field Bioassays

Beetles were trapped with custom-built flight-intercept traps (Molander et al. 2019). Briefly, the traps had two cross-vane panels with a plastic funnel suspended below them, with the spout of the funnel protruding into the collecting jar at the bottom of the trap, and a flat top-cover for protection from rain. To maximize trapping efficiency and retention of captured beetles, the panels and the inside of the funnel were painted with a polytetrafluoroethylene dispersion (60 wt% in H_2_O, Fluon^®^; Sigma-Aldrich, St. Louis, Missouri, USA), diluted 1:1 with water (Graham and Poland 2012). Propylene glycol (~0.25 L per trap) was used as a killing agent and preservative. In 2013, traps were suspended from tree branches at approximately breast height. In 2017, we used steel reinforcing bar posts to hang the traps at about the same height above ground.

Traps were deployed in linear transects, spaced ~10 m apart within replicates, with one treatment of each type per replicate assigned randomly at deployment. The traps were serviced at intervals of 2–4 wk., with treatment positions re-randomized at every visit. When collecting jars were emptied, the propylene glycol was filtered to separate the insects, and then reused. Trap samples were initially stored at 4 °C, then transferred to 70% ethanol for long-term storage after sorting their contents. Voucher specimens will be donated to the entomological collection of the Biological Museum, Lund University, Sweden.

Traps were baited with dispensers made from polyethylene zip-lock bags (Grippie^®^ Light Nr-02, 5.5 × 6.5 cm × 40 μm, b.n.t. Scandinavia AB, Arlöv, Sweden). After loading, bags were sealed and suspended with metal wire near the center of the cross-vanes of the trap without puncturing the bag. Lures were loaded with 0.5 ml of isopropanol containing different amounts of putative pheromone components (see below), whereas controls were loaded with 0.5 ml isopropanol alone.

Bioassay 1 (2013) included an isopropanol control and different amounts (mg) of racemic 2-methyl-1-butanol and racemic 3-hydroxy-2-hexanone (with the % of 2-methyl-1-butanol in relation to 3-hydroxy-2-hexanone shown in parentheses) in ratios of 0:50 (0%), 1:50 (2%), 2.5:50 (5%), 5:50 (10%), 10:50 (20%), and 50:0 (single component). The bioassays included four blocks of traps (spatial replicates) at Gö nature reserve, which were deployed between 5 May and 12 August 2013. The traps were emptied and the pheromone lures were replaced on 20 May, 31 May, 5 July, and 12 August, creating four separate sampling periods.

Bioassay 2 (2017) was similar to bioassay 1, but with a different range of ratios of 2-methyl-1-butanol to 3-hydroxy-2-hexanone, to cover the whole range that was found in the extracts of both *P. sanguineum* and *P. alni*, as follows (in mg, in 0.5 ml isopropanol): 0:50 (0%), 10:50 (20%), 25:50 (50%), 40:50 (80%), 50:0 (single component), and a control (0.5 ml isopropanol alone). The bioassays included six blocks (spatial replicates) of traps at Ecopark Hornsö that were deployed on 30 April. The traps were emptied and lures were replaced on 20 May, 11 June, 2 July, and 24 July.

Trapped cerambycids were identified using the key by Ehnström and Holmer (2007). We also examined the sex ratios of the three different species in subsets of the trap catches from bioassay 2. Males and females of the two larger species, *P. sanguineum* and *P. testaceus*, were separated based on the relative length of the antennae in relation to the body length (males have noticeably longer antennae than females), but a subset of about 20 individuals of each species were also dissected and their genitalia examined to verify that the length of the antennae was a consistently valid character for separation of the sexes. For the small species *P. alni*, we dissected all individuals in the subset and studied their genitalia.

### Statistical Analysis

The datasets from the bioassays exhibited heteroscedasticity and beetle numbers were not normally distributed. Further, trap catches were strongly influenced by trapping site (spatial replicate) and the defined annual activity periods of the beetles (temporal replicates). To account for these effects, we analyzed the data with Generalized Linear Mixed Models (GLMMs) and multiple post hoc comparisons (Supplementary Tables 1 and 2). The models compared how the response variable (catch per trap) depended on treatment (fixed factor), and included replicate (site) and trapping period (temporal replicate) as random effects. First, we fitted a GLMM to each of the six datasets using the Poisson distribution (link function: log_*e*_) and calculated the dispersion statistic (φ) for each model using Pearson residuals. Three models showed negligible over- or underdispersion (*P. sanguineum* 2013 φ = 1.14, *P. alni* 2013 φ = 0.51, *P. testaceus* 2017 φ = 1.28), with no need for model corrections. However, three of the models showed overdispersion (*P. testaceus* 2013 φ = 1.69, *P. sanguineum* 2017 φ = 9.04, and *P. alni* 2017 φ = 5.27). For these three models, we exchanged the Poisson distribution for a negative binomial distribution (link function log_*e*_). The major inferences (significant differences) remained nearly identical, with the exception that the treatment with 3-hydroxy-2-hexanone as a single component was not significantly different from the control in 2013 for *P. testaceus* when the negative binomial distribution was used (*P* = 0.069). New dispersion statistics were calculated for the models based on the negative binomial distribution (φ = 0.94, φ = 0.62 and φ = 0.86, respectively), and deemed acceptable.

After fitting the models, we compared the treatments by multiple pairwise post hoc tests using least squares means (*Tukey’s HSD test*, Supplementary Table 2). All calculations were performed in R version 3.5.1 for Windows (R Core Team 2018), with the lme4 package for GLMMs (Bates et al. 2015), and the emmeans package (Lenth 2018). Statistical significance was defined as probability values (*P*), or adjusted probability values (*P*_*a*_) for post hoc tests, of less than 0.05.

Sampling periods (temporal replicates) or individual sites (spatial replicates) where no beetles of the focal species were captured in any trap were excluded from the analyses. Further, in four cases, the control treatments did not capture any beetles at all throughout the season. In order to allow for the models to be fitted, we inserted an artificial observation of one individual to the control that belonged to the spatial and temporal replicate where the other treatments had captured the highest total number of individuals (see Supplementary Table 1). Similarly, three other treatments for *P. alni* in 2013 only contained zero observations, and we inserted an artificial observation of one individual into each of these three treatments with the same method as for the controls with only zero counts. Occasionally, stray individuals were captured outside of the main activity period of the species (one *P. alni* and one *P. testaceus* respectively in 2013; one *P. sanguineum* and nine individuals of *P. alni* in 2017, all captured with blend treatments). Because these beetles either were the only individuals captured during a whole temporal replicate, or were caught in much smaller numbers than what was observed during their main flight period respectively (*P. alni* in 2017), we excluded these captures from data analyses because their inclusion only served to extend the variance unnecessarily. The captures mentioned above also were not included in the total catches or the figures (see Results).

## Results

### Identification of Insect-Produced Volatiles

**Fig. 1:**
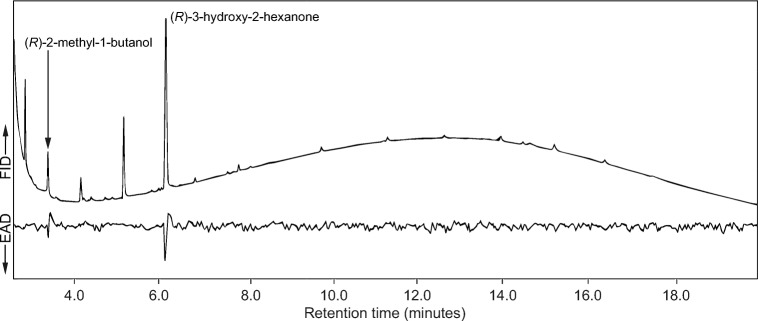
Representative coupled gas chromatography-electroantennogram detection analysis of extracts of headspace volatiles from males of *P. sanguineum*, using an antenna from a male beetle of the same species. Antennae consistently responded to two compounds in these extracts, identified as (*R*)-2-methyl-1-butanol and (*R*)-3-hydroxy-2-hexanone. Analogous responses were elicited from antennae of females when stimulated with the same extracts

**Fig. 2:**
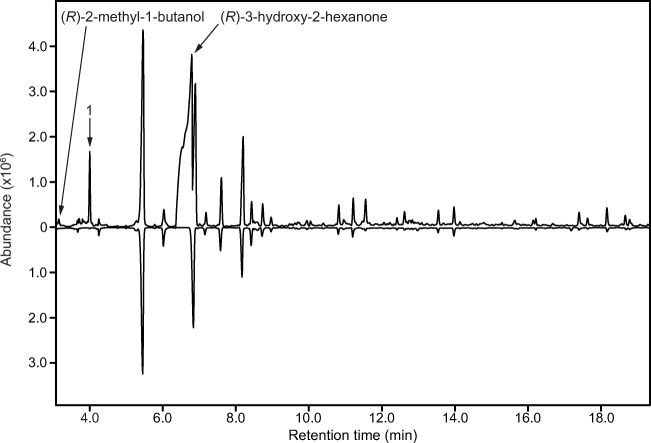
GC-MS analysis of extracts of headspace volatiles from male (top trace) and female (bottom, inverted trace) *P. sanguineum* (HP-5 MS column). Three insect-produced compounds were consistently present, and specific, to the extracts of male beetles: (*R*)-2-methyl-1-butanol, (*R*)-3-hydroxy-2-hexanone, and 2,3-hexanedione (1). The remaining compounds, present in extracts from both sexes, are system contaminants, found in the ambient air where the aerations were performed

In GC-EAD analyses, two compounds present in all headspace collections from males of *P. sanguineum* elicited responses from 5 of 7 antennae of males and 5 out of 10 antennae of females (Fig. 1). In subsequent GC-MS analyses, the two compounds were identified as 3-hydroxy-2-hexanone and 2-methyl-1-butanol (Fig. 2), and the absolute configurations of both compounds were determined to be (*R*). No other compounds in the extracts of males elicited any consistent responses from antennae of either sex. Corresponding analyses with extracts of females resulted in no consistent responses from antennae of males or females, and none of the extracts from females contained 3-hydroxy-2-hexanone or 2-methyl-1-butanol. Based on five independent collections from male beetles, (*R*)*-*3-hydroxy-2-hexanone was the major component, with (*R*)-2-methyl-1-butanol present in quantities varying from 1 to 15:100 (mean 7:100) of the major component. In addition, 2,3-hexanedione was consistently present in the headspace collections from male *P. sanguineum* at a mean ratio of 6:100 to (*R*)-3-hydroxy-2-hexanone.

**Fig. 3:**
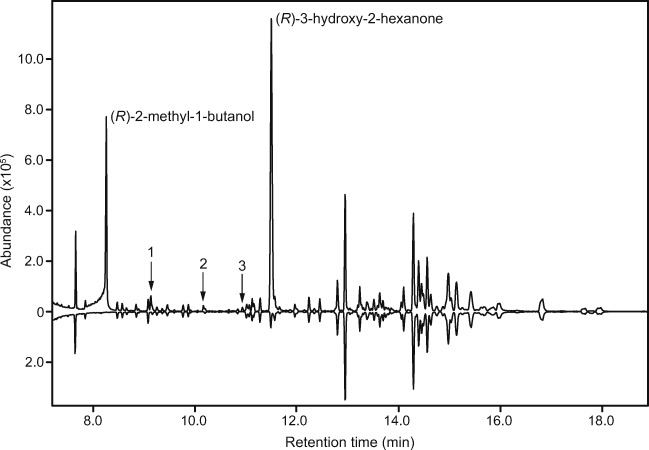
GC-MS analysis of extracts of headspace volatiles from male (top trace) and female (bottom, inverted trace) *P. alni* (HP-5 MS column). Two insect-produced compounds occurred in large quantities and were specific to the extracts of male beetles: (*R*)-2-methyl-1-butanol and (*R*)-3-hydroxy-2-hexanone. In addition, minor quantities of three other compounds were present in the extracts of male beetles, but did not occur in the corresponding extracts of females, including 2,3-hexanedione (1), 2-methyl-1-pentanol (2) and 1-hexanol (3). The remaining compounds, present in extracts from both sexes, are system contaminants, found in the ambient air where the aerations were performed (the compound in the extract from females with the same retention time as (*R*)-3-hydroxy-2-hexanone is styrene)

Analyses of headspace volatiles from *P. alni* demonstrated that males produced the same two compounds as males of *P. sanguineum*, (*R*)*-*3-hydroxy-2-hexanone and (*R*)-2-methyl-1-butanol, which were not detected in samples from females (Fig. 3). The ratio of (*R*)-2-methyl-1-butanol to (*R*)*-*3-hydroxy-2-hexanone was 70:100 and 110:100 in two extracts from males (mean ratio: 90:100). In both extracts, three additional male-specific compounds were detected in minor (2,3-hexanedione; mean ratio 5:100) or trace quantities (2-methyl-1-pentanol; mean ratio 2:100 and 1-hexanol; mean ratio 1:100). No female-specific compounds were detected in the extracts of females, nor were any compounds common to both sexes detected in any of the insect extracts, but missing in the blank control headspace collections.

No live specimens of *P. testaceus* were collected from the emergence cages, although 21 dead specimens were found when the boxes were cleaned out. Thus, headspace extracts were not available for analysis.

### Field Bioassays

**Fig. 4:**
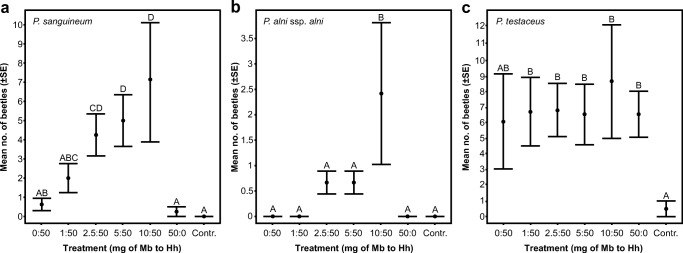
Results from field trapping of *P. sanguineum* (**a**), *P. alni* (**b**) and *P. testaceus* (**c**) in bioassay 1 (2013). The mean number of captured beetles per trap and collection date is presented for different lure treatments. The lures consisted of different quantities (mg) of racemic 2-methyl-1-butanol (Mb) and racemic 3-hydroxy-2-hexanone (Hh) in 0.5 ml of isopropanol. Controls were baited with 0.5 ml of isopropanol alone. Treatments that do not share a common letter are significantly different (adjusted *P* < 0.05) (for details, see Supplementary Table 2)

In the first bioassay (2013), a total of 172 *P. sanguineum* were captured. Neither of the individual compounds was attractive versus the control, but blends with 2-methyl-1-butanol: 3-hydroxy-2-hexanone ratios from 2.5:50 to 10:50 mg were significantly more attractive than the control or the individual compounds (Fig. 4a) (for details on all post hoc tests, see Supplementary Table 2). Further, the traps captured a total of 45 *P. alni*, and this species exhibited a similar pattern of attraction to that of *P. sanguineum*. However, only the mean catch for the 10:50 blend was significantly higher than the control (Fig. 4b). No other treatments differed significantly from each other. In contrast, *P. testaceus* exhibited a different pattern of trap catches. A total of 166 beetles were caught, and all treatments, except 3-hydroxy-2-hexanone as a single component, attracted significantly more beetles than the control, but none of the treatments were significantly different from each other (Fig. 4c).

**Fig. 5:**
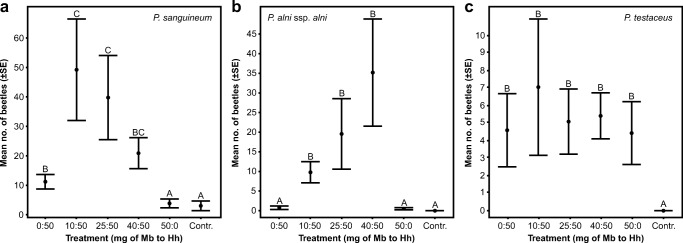
Results from field trapping of *P. sanguineum* (**a**), *P. alni* (**b**) and *P. testaceus* (**c**) in bioassay 2 (2017). The mean number of captured beetles per trap and collection date is presented for different lure treatments. The lures consisted of different quantities (mg) of racemic 2-methyl-1-butanol (Mb) and racemic 3-hydroxy-2-hexanone (Hh) in 0.5 ml of isopropanol. The controls were baited with 0.5 ml of isopropanol alone. Treatments that do not share a common letter are significantly different (adjusted *P* < 0.05) (for details, see Supplementary Table 2)

In the second bioassay (2017), both *P. sanguineum* (1528 individuals) and *P. alni* (787 individuals) were captured in much higher total numbers than in bioassay 1. *Pyrrhidium sanguineum* was significantly more attracted to all binary blends compared to the control and 2-methyl-1-butanol as a single component (Fig. 5a). The 10:50 and 25:50 blends also captured significantly more beetles than 3-hydroxy-2-hexanone as a single component, but the 40:50 blend did not differ significantly from 3-hydroxy-2-hexanone as a single component, and the three blends did not differ significantly from each other (Fig. 5a). The treatment with 3-hydroxy-2-hexanone as a single component captured significantly more beetles compared to the control or 2-methyl-1-butanol as a single component. For *P. alni*, attraction to all binary blends was significantly higher than to the control or the individual compounds, but the three blends were not significantly different from each other (Fig. 5b). The individual compounds did not differ from the control. Roughly the same number of *P. testaceus* (162 individuals) were captured in the second bioassay. Similar to bioassay 1, all treatments attracted significantly more beetles than the control, but none of the treatments differed from each other (Fig. 5c).

Examination of the sex ratios of beetles caught in bioassay 2 revealed that catches consisted of both males and females of all species at about even ratios. The sex ratio was 55% males and 45% females from a sample of 183 *P. sanguineum* (from a trap with a 25:50 blend, first count), and 38% males and 62% females from a sample of 135 *P. alni* (from a trap with a 40:50 blend, second count). For *P. testaceus*, 18 beetles from 6 traps baited with 50 mg 2-methyl-1-butanol alone were 39% males and 61% females (third count), 25 beetles from 6 traps baited with 50 mg 3-hydroxy-2-hexanone alone were 52% males and 48% females (third count), and 30 beetles from 6 traps baited with a 25:50 blend were 37% males and 63% females (third count).

## Discussion

Our study demonstrates that the male-produced aggregation-sex pheromone blends of the longhorn beetles *P. sanguineum* and *P. alni* consist of (*R*)-3-hydroxy-2-hexanone and (*R*)-2-methyl-1-butanol. The minor component, 2-methyl-1-butanol, was not reported as a component of the volatiles produced by male *P. sanguineum* in a previous study (Schröder et al. 1994; Schröder 1996). In the work presented here, we first obtained evidence for two possible pheromone components in *P. sanguineum* from GC-EAD analyses of headspace volatiles, and then followed up with identification and field testing of the two compounds, demonstrating that (*R*)-2-methyl-1-butanol was indeed a key pheromone component for *P. sanguineum*, in addition to (*R*)-3-hydroxy-2-hexanone. In the previous work, Schröder et al. (1994) reported (*R*)-3-hydroxy-2-hexanone as the main pheromone component, but the extracts of males also contained trace quantities of (2*R*,3*R*)-2,3-hexanediol and (2*S*,3*R*)-2,3-hexanediol, in addition to 1-butanol, 2,3-hexanedione and both enantiomers of 2-hydroxy-3-hexanone (Fettköther 1995; Schröder 1996). The latter two compounds were likely artefacts formed by degradation of (*R*)-3-hydroxy-2-hexanone under the GC conditions used (Millar and Hanks 2017; Schröder et al. 1994), and we too noted the presence of these compounds during our work (depending on the temperatures used for GC analyses). However, we did not detect the hexanediols or 1-butanol in our extracts. In wind tunnel experiments, unmated females of *P. sanguineum* (but not males) had been shown to be significantly attracted to a synthetic four-component blend of (*R*)-3-hydroxy-2-hexanone, the 2,3-hexanediols and 2,3-hexanedione, as well as (*R*)-3-hydroxy-2-hexanone as a single compound, respectively (Fettköther 1995; Schröder 1996). The most important compound for attraction in the wind tunnel assays appeared to be (*R*)-3-hydroxy-2-hexanone, as 2,3-hexanedione alone and a mixture of the two hexanediols, were either not (2,3-hexanedione), or only weakly attractive (the mixture of hexanediols) to the unmated females (Fettköther 1995). 2-Hydroxy-3-hexanone and 1-butanol were not tested in the wind tunnel experiments (Fettköther 1995). Significant attraction to 3-hydroxy-2-hexanone as a single compound was also shown in one of our bioassays, but in general *P. sanguineum* was significantly more attracted to blends of 3-hydroxy-2-hexanone and 2-methyl-1-butanol than to either compound alone. This result is similar to the observations from two independent field bioassays conducted in Hungary (Imrei et al. 2013) and Sweden (IBW et al., unpubl. data), where single compounds were not significantly attractive to this species. The potential importance of the 2,3-hexanediols and 1-butanol for attraction remains unclear, but they are evidently not needed to achieve significant attraction.

In contrast to the other two species, for which the single components elicited no significant (or comparatively weak) attraction, *P. testaceus* was attracted to both 2-methyl-1-butanol and 3-hydroxy-2-hexanone as single compounds (although the response to the hydroxyketone alone was not significant in 2013), and to all the blends of the two compounds (total range of ratios 2:100 to 80:100). No apparent additive or synergistic effects were obtained from blending the two components, contrary to what was observed for the two other species. Attraction of *P. testaceus* to 2-methyl-1-butanol is consistent with its previously reported pheromone chemistry, because males have been shown to produce (*R*)-2-methyl-1-butanol (Hanks et al. 2019), but the response to the hydroxyketone alone, which this species does not produce, is more difficult to explain. However, the observation is far from unique. Sweeney et al. (2014) reported significant attraction of *P. testaceus* to traps baited with a combination of the hydroxyketone and ethanol, and in recent studies in Poland and Italy, *P. testaceus* was significantly attracted to “multi-lure” blends that included racemic 3-hydroxy-2-hexanone, but not 2-methyl-1-butanol (although additional compounds were present in the multi-lures; Flaherty et al. 2019; Rassati et al. 2019). Field studies conducted in the eastern USA, where *P. testaceus* was introduced many decades ago (Swift and Ray 2010), have revealed inconsistent responses to the two compounds. For example, adults of *P. testaceus* were attracted in significant numbers to traps baited with 3-hydroxy-2-hexanone alone during bioassays that lacked a 2-methyl-1-butanol treatment, but only the latter compound was attractive when both compounds were presented as single compound treatments in other bioassays (Hanks and Millar 2013; Hanks et al. 2018, 2019; Millar et al. 2018). Other field trials conducted in the eastern USA, with traps baited with blends of cerambycid pheromones that included 2-methyl-1-butanol and 3-hydroxy-2-hexanone (Handley et al. 2015; Hanks and Millar 2013), have also demonstrated significant attraction of *P. testaceus* to these blends. More comprehensive studies are needed to determine if *P. testaceus* has a preference for its own pheromone component or the heterospecific hydroxyketone, and how preference might be affected by different conditions.

In a wider perspective, insect sex or aggregation-sex pheromones are commonly assumed to be species-specific signals bringing individuals of the opposite sex together for mating (Cardé 2014). In cerambycid beetles, however, there appears to be a range of specificity, from rather promiscuous cross-attraction of a number of species to a single component or blend, to attraction of only a single species (Millar and Hanks 2017). For the species studied here, the potential for cross-attraction among species is considerable, based on the identified pheromone blends and observed trap catches. Furthermore, both 3-hydroxy-2-hexanone and 2-methyl-1-butanol are common pheromone components that are utilized by many other species in the subfamily Cerambycinae (Hanks and Millar 2016). Thus, in field bioassays, attraction of several longhorn beetle species to a single synthetic pheromone component, or a blend of components, is a common phenomenon (Mitchell et al. 2015). A possible explanation for the attraction of *P. testaceus* to the heterospecific pheromone component 3-hydroxy-2-hexanone is that there may be adaptive reasons for eavesdropping on the pheromonal signals of other guild members. For example, heterospecific signals could be exploited as cues to locate relatively scarce and ephemeral breeding substrates (see Hanks et al. 2007; Molander and Larsson 2018). Cross-attraction between heterospecific blends could be exacerbated by the fact that many cerambycine beetles appear to be relatively indifferent to the presence of the opposite enantiomers, with racemic mixtures often being as attractive as the insect-produced enantiomers (e.g., Hanks et al. 2019).

In our study, all three species were attracted to the same compounds, and differences in the blend ratios of 3-hydroxy-2-hexanone and 2-methyl-1-butanol only appear to constitute weak barriers to cross-attraction among the species. However, accumulating evidence suggests that many cerambycid species have relatively specific seasonal and diel activity patterns (Meier et al. 2019; Mitchell et al. 2015), only producing or responding to pheromones during specific time windows, providing additional mechanisms by which cross-attraction can be avoided, even among species with identical pheromones. Such subtleties may be obscured in trials with pheromone-baited traps, the lures of which release pheromones continuously, rather than only during discrete time windows. In fact, some separation of our species may occur due to different circadian rhythms because *P. sanguineum* and *P. alni* are primarily diurnal, whereas *P. testaceus* is predominantly crepuscular and/or nocturnal (LMH, MAM pers. obs.). The crepuscular/nocturnal habits of the latter species may also explain why we did not observe live *P. testaceus* in the emergence boxes, which were only inspected during the day. The seasonal phenologies of the three species are also partially separated, but the degree of overlap is substantial, particularly between *P. sanguineum* and *P. alni*. Typically, *P. sanguineum* emerges in late April in Sweden, with an activity peak around 20th May, whereas *P. alni* starts to emerge after the first week of May, with activity peaking in the first week of June. Similarly, *P. testaceus* starts to emerge in early June and has an activity peak in the first week of July (Lindhe et al. 2010). Furthermore, the substrates used by the three species in this study are superficially similar, but could provide some degree of separation at a fine scale. *Phymatodes alni* utilizes thin twigs (1–2.5 cm ø) for oviposition, whereas *P. sanguineum* and *P. testaceus* primarily use somewhat larger branches and tree trunks (ø > 5 cm) (Ehnström and Holmer 2007; MAM pers. obs.). In addition, whereas *P. sanguineum* and *P. alni* breed almost exclusively in oak in Sweden, *P. testaceus* will readily colonize several other deciduous trees, although oak is the primary host (Ehnström and Holmer 2007). *Phymatodes testaceus* also appears to prefer more desiccated wood and bark compared to the two other species (Ehnström and Holmer 2007).

A common feature in male-produced aggregation-sex pheromone systems is that they are frequently centered on attraction towards valuable breeding substrates, whose kairomonal cues combine with the pheromone to form an integral part of the overall attractive signal (Landolt and Philips 1997; Schlyter and Birgersson 1999). This phenomenon has also been observed with a number of cerambycid species, where the male-produced aggregation-sex pheromones act synergistically with host plant volatiles (e.g., Collignon et al. 2016; many *Monochamus* spp., reviewed in Hanks and Millar 2016). It remains to be determined whether host volatiles may act additively or synergistically with our study species. Some or all of the above factors may contribute to reproductive isolation among these species, and further research is needed to elucidate the mechanisms involved and their relative importance.

For the purpose of monitoring saproxylic insect species as indicators of the effects of forest management and landscape change, it would be beneficial to monitor a broad range of species with different niche requirements, such as different host tree species, different dimensions of wood substrates, and different decomposition stages of woody material. At present, the lack of effective monitoring tools remains the most severe bottleneck for implementing large-scale conservation monitoring of endangered saproxylic insects. Thus, the fact that compounds are often shared, and the indifference to the presence of the “unnatural” enantiomer of a pheromone component, is fortuitous from a practical perspective, and should be viewed as an advantage, because it allows the simultaneous monitoring of several target species with the same lure and trap. For example, in the present study we identified a blend that attracts three cerambycid species representing different, but overlapping, substrate niches and host tree specificities among ephemeral substrates associated with fresh deadwood resources of oak. As suggested by the bioassay data from 2017, a suitable trade-off to monitor these species efficiently appears to be a 50:100 blend of the racemates of 2-methyl-1-butanol and 3-hydroxy-2-hexanone, because all three species were significantly attracted to this blend. With pheromone-based monitoring as an effective sampling tool, these species may constitute cost-efficient model species as indicators of variation in the availability of fresh deadwood resources in managed forests. This in turn will provide an assessment of the suitability of different management regimes for conserving the deadwood resources required as host material by these and many other saproxylic species.

## Electronic supplementary material

ESM 1 (DOCX 104 kb)
