# Supplementary material for: Common Cerambycid Pheromone Components as Attractants for Longhorn Beetles (Cerambycidae) Breeding in Ephemeral Oak Substrates in Northern Europe
Source: J Chem Ecol. 2019 Jun 28;45(7):537–48. doi: 10.1007/s10886-019-01082-4 (PMC6661259; doi:10.1007/s10886-019-01082-4)
Supplement: Supplementary file 1 — (DOCX 104 kb) [file 10886_2019_1082_MOESM1_ESM.docx]

**Supplementary material**

for

# **Common Cerambycid Pheromone Components as Attractants for Longhorn Beetles (Cerambycidae) Breeding in Ephemeral Oak Substrates in Northern Europe**

*Journal of Chemical Ecology*

**Mikael A. Molander^*^, Inis B. Winde, Joseph Burman,**

**Franklin N. Nyabuga, Tobias U. T. Lindblom, Lawrence M. Hanks, Jocelyn G. Millar, Mattias C. Larsson**

*Corresponding author: Unit of Chemical Ecology, Department of Plant Protection Biology, Swedish University of Agricultural Sciences, Box 102, Sundsvägen 14, 230 53 Alnarp, Sweden, [mikael.molander@slu.se](mailto:mikael.molander@slu.se)

**Description of content**

The supplement contains the results from the statistical analysis (GLMMs and post hoc tests) of the attraction of the three cerambycid species (*Pyrrhidium sanguineum*, *Phymatodes alni*, and *Phymatodes testaceus*) to different pheromone lures in two field bioassays (2013 and 2017), performed in southeastern Sweden during identification of the species’ aggregation-sex pheromones.

**Table S1** Results of GLMMs comparing the number of captured cerambycids of three species (*P. sanguineum*, *P. alni*, and *P. testaceus*) in traps baited with synthetic pheromone compounds in two independent field bioassays performed in southern Sweden (2013 and 2017). Each model examines the effect of six (2013) or five (2017) different lure treatments of the racemates of 2-methyl-1-butanol and 3-hydroxy-2-hexanone (formulated in 0.5 ml isopropanol) to a control (intercept) (0.5 ml of isopropanol alone). Two treatments tested the compounds as single components (50 mg 2-methyl-1-butanol and 50 mg 3-hydroxy-2-hexanone per lure respectively), and four or three treatments consisted of blends with a variable quantity of 2-methyl-1-butanol (for instance 10, 25, and 40 mg per lure in 2017) to that of the hydroxyketone (50 mg racemic 3-hydroxy-2-hexanone per lure in all blends). Treatments are denoted in the format of 2-methyl-1-butanol (mg) to 3-hydroxy-2-hexanone (mg). The link function with log*e* was used for all six models

| **Dataset, distribution** | **Fixed effects** | **Estimate^a^** | **SE** | ***Z*** | ***P*(>\|*z*\|)** |
| --- | --- | --- | --- | --- | --- |
| 2013 *P. sanguineum*, Poisson^b^ | Contr. (intercept) | -2.35 | 1.09 | -2.16 | **0.031** |
|  | 0:50 | 0.69 | 1.21 | 0.57 | 0.568 |
|  | 50:0 | 1.61 | 1.09 | 1.48 | 0.138 |
|  | 1:50 | 2.77 | 1.02 | 2.72 | **0.007** |
|  | 2.5:50 | 3.52 | 1.01 | 3.51 | **<0.001** |
|  | 5:50 | 3.69 | 1.00 | 3.68 | **<0.001** |
|  | 10:50 | 4.03 | 1.00 | 4.03 | **<0.001** |
|  |  |  |  |  |  |
| 2013 *P. alni*,  Poisson^b, c^ | Contr. (intercept) | -3.12 | 1.12 | -2.78 | **0.005** |
|  | 0:50 | -1.13 | 1.41 | 0.01 | 1.000 |
|  | 50:0 | -1.13 | 1.41 | 0.01 | 1.000 |
|  | 1:50 | -1.13 | 1.41 | 0.01 | 1.000 |
|  | 2.5:50 | 2.08 | 1.06 | 1.96 | **0.049** |
|  | 5:50 | 2.08 | 1.06 | 1.96 | **0.049** |
|  | 10:50 | 3.37 | 1.02 | 3.31 | **<0.001** |
|  |  |  |  |  |  |
| 2013 *P. testaceus*,  Neg. Bin. | Contr. (intercept) | -1.99 | 1.14 | -1.74 | 0.083 |
|  | 0:50 | 2.36 | 0.83 | 2.83 | **0.005** |
|  | 50:0 | 2.63 | 0.83 | 3.17 | **0.002** |
|  | 1:50 | 2.64 | 0.83 | 3.18 | **0.005** |
|  | 2.5:50 | 2.78 | 0.83 | 3.34 | **0.002** |
|  | 5:50 | 2.55 | 0.83 | 3.07 | **<0.001** |
|  | 10:50 | 2.72 | 0.82 | 3.31 | **<0.001** |
|  |  |  |  |  |  |
| 2017 *P. sanguineum*, Neg. Bin. | Contr. (intercept) | 0.79 | 0.40 | 1.99 | **0.047** |
|  | 0:50 | 1.55 | 0.31 | 4.93 | **<0.001** |
|  | 50:0 | 0.33 | 0.33 | 1.01 | 0.314 |
|  | 10:50 | 2.77 | 0.30 | 9.17 | **<0.001** |
|  | 25:50  **Table S1 continued:** | 2.51 | 0.30 | 8.37 | **<0.001** |
|  | 40:50 | 2.08 | 0.31 | 6.81 | **<0.001** |
|  |  |  |  |  |  |
| 2017 *P. alni*,  Neg. Bin.^b^ | Contr. (intercept) | -3.27 | 1.27 | -2.57 | **0.010** |
|  | 0:50 | 2.15 | 1.19 | 1.81 | 0.071 |
|  | 50:0 | 1.77 | 1.21 | 1.46 | 0.144 |
|  | 10:50 | 5.14 | 1.14 | 4.50 | **<0.001** |
|  | 25:50 | 5.54 | 1.14 | 4.88 | **<0.001** |
|  | 40:50 | 6.10 | 1.13 | 5.37 | **<0.001** |
|  |  |  |  |  |  |
| 2017 *P. testaceus*, Poisson^b^ | Contr. (intercept) | -3.78 | 1.17 | -3.22 | **0.001** |
|  | 0:50 | 3.33 | 1.00 | 3.34 | **<0.001** |
|  | 50:0 | 3.30 | 1.00 | 3.30 | **<0.001** |
|  | 10:50 | 3.76 | 0.99 | 3.79 | **<0.001** |
|  | 25:50 | 3.43 | 1.00 | 3.45 | **<0.001** |
|  | 40:50 | 3.50 | 1.00 | 3.51 | **<0.001** |

^a^Estimates are presented on the log*_e_* scale

^b^An artificial observation of one individual was inserted into the control to allow for the model to be fitted

^c^An artificial observation of one individual was inserted into each of the three treatments 0:50, 50:0, and

1:50 to allow for the model to be fitted

**Table S2** Least squares means analysis (following GLMMs, see Supplementary Table 1) of trap catches from two independent field bioassays of pheromone compounds for three species of cerambycids, performed in southern Sweden (2013, 2017). *Tukey’s HSD test* was used for the multiple post hoc comparisons of the numbers of captured beetles (*P. sanguineum*, *P. alni*, and *P. testaceus*). Two treatments tested the compounds as single components (50 mg 2-methyl-1-butanol and 50 mg 3-hydroxy-2-hexanone per lure respectively in 0.5 ml isopropanol), and four (2013) or three (2017) treatments consisted of blends with a variable quantity of 2-methyl-1-butanol (for instance 10, 25, and 40 mg per lure) to that of the hydroxyketone (50 mg racemic 3-hydroxy-2-hexanone per lure in all blends), dissolved in 0.5 ml of isopropanol. The control consisted of 0.5 ml isopropanol alone. Treatments are denoted in the format of 2-methyl-1-butanol (mg) to 3-hydroxy-2-hexanone (mg)

| **Dataset** | **Comparison** | **Estimate^a^** | **SE** | ***Z*** | ***P_a_*(>\|*z*\|)^b^** |
| --- | --- | --- | --- | --- | --- |
| 2013  *P. sanguineum*^c^ | Contr. ‒ 0:50 | 0.20 | 0.22 | -1.48 | 0.755 |
|  | Contr. ‒ 50:0 | 0.50 | 0.61 | -0.57 | 0.998 |
|  | Contr. ‒ 1:50 | 0.06 | 0.06 | -2.72 | 0.094 |
|  | Contr. ‒ 2.5:50 | 0.03 | 0.03 | -3.51 | **0.008** |
|  | Contr. ‒ 5:50 | 0.03 | 0.03 | -3.68 | **0.004** |
|  | Contr. ‒ 10:50 | 0.02 | 0.02 | -4.03 | **0.001** |
|  | 50:0 ‒ 0:50 | 0.40 | 0.33 | -1.11 | 0.927 |
|  | 50:0 ‒ 1:50 | 0.13 | 0.09 | -2.80 | 0.076 |
|  | 50:0 ‒ 2.5:50 | 0.06 | 0.04 | -3.93 | **0.002** |
|  | 50:0 ‒ 5:50 | 0.05 | 0.04 | -4.18 | **0.001** |
|  | 50:0 ‒ 10:50 | 0.04 | 0.03 | -4.68 | **0.001** |
|  | 0:50 ‒ 1:50 | 0.31 | 0.16 | -2.29 | 0.246 |
|  | 0:50 ‒ 2.5:50 | 0.15 | 0.07 | -4.04 | **0.001** |
|  | 0:50 ‒ 5:50 | 0.13 | 0.06 | -4.43 | **<0.001** |
|  | 0:50 ‒ 10:50 | 0.09 | 0.04 | -5.23 | **<0.001** |
|  | 1:50 ‒ 2.5:50 | 0.47 | 0.14 | -2.51 | 0.155 |
|  | 1:50 ‒ 5:50 | 0.40 | 0.12 | -3.13 | **0.029** |
|  | 1:50 ‒ 10:50 | 0.29 | 0.08 | -4.46 | **<0.001** |
|  | 2.5:50 ‒ 5:50 | 0.85 | 0.20 | -0.70 | 0.992 |
|  | 2.5:50 ‒ 10:50 | 0.61 | 0.13 | -2.32 | 0.235 |
|  | 5:50 ‒ 10:50 | 0.71 | 0.15 | -1.64 | 0.655 |
|  |  |  |  |  |  |
| 2013  *P. alni*^c, d^ | Contr. ‒ 0:50 | 0.01 | 1.14 | 0.01 | 1.000 |
|  | Contr. ‒ 50:0 | 0.01 | 1.14 | 0.01 | 1.000 |
|  | Contr. ‒ 1:50 | 0.01 | 1.14 | 0.01 | 1.000 |
|  | Contr. ‒ 2.5:50 | -2.08 | 1.06 | -1.96 | 0.448 |
|  | Contr. ‒ 5:50 | -2.08 | 1.06 | -1.96 | 0.448 |
|  | Contr. ‒ 10:50 | -3.37 | 1.02 | -3.31 | **0.023** |
|  | 50:0 ‒ 0:50  **Table S2 continued:** | 0.01 | 1.14 | 0.01 | 1.000 |
|  | 50:0 ‒ 1:50 | 0.01 | 1.14 | 0.01 | 1.000 |
|  | 50:0 ‒ 2.5:50 | -2.08 | 1.06 | -1.96 | 0.448 |
|  | 50:0 ‒ 5:50 | -2.08 | 1.06 | -1.96 | 0.448 |
|  | 50:0 ‒ 10:50 | -3.37 | 1.02 | -3.31 | **0.023** |
|  | 0:50 ‒ 1:50 | 0.01 | 1.14 | 0.01 | 1.000 |
|  | 0:50 ‒ 2.5:50 | -2.08 | 1.06 | -1.96 | 0.448 |
|  | 0:50 ‒ 5:50 | -2.08 | 1.06 | -1.96 | 0.448 |
|  | 0:50 ‒ 10:50 | -3.37 | 1.02 | -3.31 | **0.023** |
|  | 1:50 ‒ 2.5:50 | -2.08 | 1.06 | -1.96 | 0.448 |
|  | 1:50 ‒ 5:50 | -2.08 | 1.06 | -1.96 | 0.448 |
|  | 1:50 ‒ 10:50 | -3.37 | 1.02 | -3.31 | **0.023** |
|  | 2.5:50 ‒ 5:50 | 0.01 | 1.14 | 0.01 | 1.000 |
|  | 2.5:50 ‒ 10:50 | -1.29 | 0.40 | -3.23 | **0.030** |
|  | 5:50 ‒ 10:50 | -1.29 | 0.40 | -3.23 | **0.030** |
|  |  |  |  |  |  |
| 2013  *P. testaceus* | Contr. ‒ 0:50 | 0.07 | 0.06 | -3.17 | **0.026** |
|  | Contr. ‒ 50:0 | 0.10 | 0.08 | -2.83 | 0.069 |
|  | Contr. ‒ 1:50 | 0.07 | 0.06 | -3.18 | **0.025** |
|  | Contr. ‒ 2.5:50 | 0.06 | 0.05 | -3.34 | **0.015** |
|  | Contr. ‒ 5:50 | 0.08 | 0.07 | -3.07 | **0.035** |
|  | Contr. ‒ 10:50 | 0.07 | 0.05 | -3.31 | **0.017** |
|  | 50:0 ‒ 0:50 | 1.32 | 0.63 | 0.57 | 0.998 |
|  | 50:0 ‒ 1:50 | 0.99 | 0.47 | -0.02 | 1.000 |
|  | 50:0 ‒ 2.5:50 | 0.86 | 0.41 | -0.32 | 1.000 |
|  | 50:0 ‒ 5:50 | 1.09 | 0.51 | 0.18 | 1.000 |
|  | 50:0 ‒ 10:50 | 0.91 | 0.42 | -0.20 | 1.000 |
|  | 0:50 ‒ 1:50 | 0.75 | 0.35 | -0.61 | 0.997 |
|  | 0:50 ‒ 2.5:50 | 0.66 | 0.32 | -0.88 | 0.976 |
|  | 0:50 ‒ 5:50 | 0.83 | 0.39 | -0.41 | 1.000 |
|  | 0:50 ‒ 10:50 | 0.69 | 0.31 | -0.81 | 0.984 |
|  | 1:50 ‒ 2.5:50 | 0.87 | 0.41 | -0.29 | 1.000 |
|  | 1:50 ‒ 5:50 | 1.10 | 0.51 | 0.20 | 1.000 |
|  | 1:50 ‒ 10:50 | 0.92 | 0.42 | -0.18 | 1.000 |
|  | 2.5:50 ‒ 5:50 | 1.26 | 0.60 | 0.49 | 1.000 |
|  | 2.5:50 ‒ 10:50 | 1.06 | 0.49 | 0.21 | 1.000 |
|  | 5:50 ‒ 10:50 | 0.83 | 0.38 | -0.39 | 1.000 |
|  |  |  |  |  |  |
| 2017  *P. sanguineum* | Contr. ‒ 0:50 | 0.21 | 0.07 | -4.93 | **<0.001** |
|  | Contr. ‒ 50:0 | 0.72 | 0.24 | -1.01 | 0.916 |
|  | Contr. ‒ 10:50  **Table S2 continued:** | 0.06 | 0.02 | -9.17 | **<0.001** |
|  | Contr. ‒ 25:50 | 0.08 | 0.02 | -8.37 | **<0.001** |
|  | Contr. ‒ 40:50 | 0.12 | 0.04 | -6.81 | **<0.001** |
|  | 0:50 ‒ 50:0 | 0.30 | 0.09 | -4.09 | **<0.001** |
|  | 0:50 ‒ 10:50 | 0.09 | 0.03 | -8.49 | **<0.001** |
|  | 0:50 ‒ 25:50 | 0.11 | 0.03 | -7.62 | **<0.001** |
|  | 0:50 ‒ 40:50 | 0.17 | 0.05 | -6.01 | **<0.001** |
|  | 50:0 ‒ 10:50 | 0.30 | 0.08 | -4.83 | **<0.001** |
|  | 50:0 ‒ 25:50 | 0.38 | 0.10 | -3.78 | **0.002** |
|  | 50:0 ‒ 40:50 | 0.59 | 0.15 | -2.06 | 0.311 |
|  | 10:50 ‒ 25:50 | 1.30 | 0.31 | 1.09 | 0.884 |
|  | 10:50 ‒ 40:50 | 1.99 | 0.49 | 2.80 | 0.058 |
|  | 25:50 ‒ 40:50 | 1.53 | 0.38 | 1.72 | 0.516 |
|  |  |  |  |  |  |
| 2017  *P. alni*^c^ | Contr. ‒ 0:50 | 0.17 | 0.21 | -1.46 | 0.688 |
|  | Contr. ‒ 50:0 | 0.12 | 0.14 | -1.81 | 0.460 |
|  | Contr. ‒ 10:50 | 0.01 | 0.01 | -4.50 | **<0.001** |
|  | Contr. ‒ 25:50 | 0.01 | 0.01 | -4.88 | **<0.001** |
|  | Contr. ‒ 40:50 | 0.01 | 0.01 | -5.37 | **<0.001** |
|  | 0:50 ‒ 50:0 | 0.69 | 0.51 | -0.51 | 0.996 |
|  | 0:50 ‒ 10:50 | 0.03 | 0.02 | -5.15 | **<0.001** |
|  | 0:50 ‒ 25:50 | 0.02 | 0.02 | -5.86 | **<0.001** |
|  | 0:50 ‒ 40:50 | 0.01 | 0.01 | -6.72 | **<0.001** |
|  | 50:0 ‒ 10:50 | 0.05 | 0.03 | -4.98 | **<0.001** |
|  | 50:0 ‒ 25:50 | 0.03 | 0.02 | -5.65 | **<0.001** |
|  | 50:0 ‒ 40:50 | 0.02 | 0.01 | -6.61 | **<0.001** |
|  | 10:50 ‒ 25:50 | 0.67 | 0.31 | -0.87 | 0.955 |
|  | 10:50 ‒ 40:50 | 0.38 | 0.18 | -2.06 | 0.310 |
|  | 25:50 ‒ 40:50 | 0.57 | 0.26 | -1.23 | 0.824 |
|  |  |  |  |  |  |
| 2017  *P. testaceus*^c^ | Contr. ‒ 0:50 | 0.04 | 0.04 | -3.30 | **0.013** |
|  | Contr. ‒ 50:0 | 0.04 | 0.04 | -3.34 | **0.011** |
|  | Contr. ‒ 10:50 | 0.02 | 0.02 | -3.79 | **0.002** |
|  | Contr. ‒ 25:50 | 0.03 | 0.03 | -3.45 | **0.008** |
|  | Contr. ‒ 40:50 | 0.03 | 0.03 | -3.51 | **0.006** |
|  | 0:50 ‒ 50:0 | 0.96 | 0.26 | -0.14 | 1.000 |
|  | 0:50 ‒ 10:50 | 0.63 | 0.15 | -1.93 | 0.383 |
|  | 0:50 ‒ 25:50 | 0.87 | 0.23 | -0.54 | 0.995 |
|  | 0:50 ‒ 40:50 | 0.82 | 0.21 | -0.79 | 0.970 |
|  | 50:0 ‒ 10:50 | 0.65 | 0.16 | -1.80 | 0.465 |
|  |  |  |  |  |  |
|  | 50:0 ‒ 25:50  **Table S2 continued:** | 0.90 | 0.23 | -0.40 | 0.999 |
|  | 50:0 ‒ 40:50 | 0.85 | 0.21 | -0.65 | 0.987 |
|  | 10:50 ‒ 25:50 | 1.39 | 0.32 | 1.42 | 0.718 |
|  | 10:50 ‒ 40:50 | 1.30 | 0.30 | 1.17 | 0.853 |
|  | 25:50 ‒ 40:50 | 0.94 | 0.23 | 0.26 | 0.999 |

^a^Estimates are presented back-transformed from the log*_e_* scale

^b^*Pa* denotes the adjusted probability

^c^An artificial observation of one individual was inserted into the control to allow for the initial GLMM

model to be fitted

^d^An artificial observation of one individual was inserted into each of the three treatments 0:50, 50:0, and

1:50 to allow for the initial GLMM model to be fitted
